# Antireduction: an ancient strategy fit for future

**DOI:** 10.1042/BSR20160085

**Published:** 2016-08-16

**Authors:** Petra Maria Becker

**Affiliations:** * IEZ–Institute for Ethnobotany and Zoopharmacognosy, Rijksstraatweg 158, 6573 DG Beek-Ubbergen, The Netherlands

**Keywords:** antioxidants, antiradicals, antireductants, carotenoids, catechin, dihydrogen toxicity, electro-biosynthesis, flavonoids, food, fumarate, health, industrial production, metabolism, methane mitigation, multifunctionality, redox biochemistry, redox homoeostasis, reductive stress, resveratrol

## Abstract

While antioxidants are on everyone's lips, antireductants are their much less-known counterparts. Following an antioxidant's definition, an antireductant prevents the chemical reduction of another compound by undergoing reduction itself. Antireductants have been traced back as far as the origin of life, which they facilitated by removal of atmospheric dihydrogen, H_2_. Moreover, as electron acceptors, antireductants equipped the first metabolic pathways, enabling lithoautotrophic microbial growth. When the Earth's atmosphere became more oxidizing, certain antireductants revealed their Janus-face by acting as antioxidants. Both capacities, united in one compound, were detected in primary as well as plant secondary metabolites. Substantiated by product identification, such antireductants comprise antiradicals (e.g. carotenoids) up to diminishers of ruminal methane emission (e.g. fumarate, catechin or resveratrol). Beyond these Janus-faced, multifunctional compounds, the spectrum of antireductants extends to pure electron-attractors (e.g. atmospheric triplet oxygen, O_2_, for plant root and gut protection). Current and prospective fields of antireductant application range from health promotion over industrial production to environmental sustainability.

## INTRODUCTION

Antioxidants are part of common parlance, whereas much less is commonly known about antireductants. Although antioxidants are not the scope of this review, they shall be covered in short to give an idea about the position of antireductants as compared with antioxidants. Currently, antioxidants form a very diverse group of compounds with different mechanisms of action. Under oxidizing conditions, antioxidants limit damage to living cells [1,2], prevent rancidity, deterioration or discolouration of food [3,4], protect metals from corrosion [5], avert thickening and acidification of fuels and lubricants [6], impede embrittlement of natural and synthetic rubber [7,8] and stabilize polyolefins, such as polypropylene [9,10]. Even in purely biological contexts, the term antioxidant is comprehensively used for quite different substances, such as for radical scavengers, for inhibitors of photosensitized oxidation, for quenchers of singlet oxygen, for inactivators of peroxides, for metal-chelators, and even for enzymes such as superoxide-dismutase and catalase. Antioxidation is extensively discussed and reviewed in literature in terms of antioxidant activities [4,11,12], methods available for activity determination [13–16] and benefits and adverse effects of antioxidants [4,11,12,15,17].

Antireductant research is likewise conducted in multiple disciplines. The term ‘antireductant’ was already used in 1929 by Chambers et al. [18] for an oxazine dye with a low standard reduction potential, which–unlike dyes with similar reduction potentials–was reduced in cytoplasm of echinoderm eggs. Scott [7] in 1965 used the designation ‘hydrogen acceptor antioxidants’ for antireductants employed under oxygen (O_2_)-deficient conditions in polymer technology. Gilbert [19] in 1968 referred to ‘antireductant mechanisms’ for protection of first life forms against atmospheric dihydrogen (H_2_). By one of the earliest definitions of an antioxidant, it is a molecule that limits the oxidation of another compound by undergoing oxidation itself [20,21]. In analogy to this definition, an antireductant is a molecule, which prevents or inhibits the reduction of another compound by being reduced itself [22,23]. Thus, antireductants are electron acceptors, or hydrogen sinks (Table 1). Compounds such as hydroquinone [24] or vitamin E [25] were among the first molecules recognized as antioxidants. Just as melatonin [25] or resveratrol [26], they are reducing-agents, or electron donors, and hence form paradigms of antioxidants in terms of the definition given above. In donor-acceptor maps, these reduced compounds were classified as bad electron-accepting antireductants [22,26]; although in the case of resveratrol, this has been refuted for intestinal conditions [27]. But then, the course of a reaction depends on the setting in which it is supposed to take place. Nonetheless, defining antioxidants as electron donors, and antireductants as electron acceptors implies opposite reactivities. Then again, antioxidation and antireduction are not necessarily opposing each other, because both aim at damage control [8,12,22]. Especially *in vivo*, most biomolecules are multifunctional [1,2,27,28], and a balanced presence of both antioxidants and antireductants seems beneficial to promote health and minimize adverse reactions [12,29].

**Table 1:** Overview of antireductants, and their protective roles in diverse sectors (with a focus on biogenic compounds) *Antireductant *sensu lato*.

Sector	Field	Purpose	Mechanism	Antireductants	References
Health	Normal body function in mammals	Natural protection from superoxide radical anion (produced in cells and blood vessels) to prevent uncontrolled reactions	Oxidation or dismutation of superoxide anion by antireductants	Fe(III) cytochrome *c*, superoxide-dismutase*	[46,47,68]
	Reductive stress (e.g. from overnutrition)	Natural protection from reductive stress, manifested as increased dissimilatory NADH:NAD^+^ ratio, and prevention of oxygen radical formation	Sacrificial reduction of e.g. human serum albumin (as endogenous antireductant)	Eicosapentanoic acid, human serum albumin, *S*-adenosylmethionine, betaine, carnitine, cholines	[48–50]
	Reductive stress (induced by DTT as reducing agent in yeast)	Natural protection of protein synthesis by prevention of ribosomal protein aggregation (caused by DTT) in the endoplasmic reticulum (ER)	Binding of misassembled proteins	ER chaperone activity of Tsa1*	[51]
	Protein damage control and clearance	Natural protection from protein damage caused by reductive stress	Degradation of redox-damaged proteins via autophagy-lysosomal pathway	Concerted action of KEAP1–NRF2 pathway and autophagy*	[29,51]
	Phases of hypoxia	(Cardio)protective effect during reductive stress caused by oxygen depletion	Less necrotic damage, due to functioning of (exogenous) fumarate as H_2_-sink competitive to lactic acid generation under anoxic conditions	Fumarate	[74]
	Ulcerative colitis	Natural protection of colonocytes from hydrogen sulfide toxicity	Oxidation of hydrogen sulfide to thiosulfate and sulfate	ETC components, with O_2_ as final electron acceptor	[45]
Food	Wheat bread production	Improved bread cohesiveness by antireductant as flour additive	Oxidative disulfide formation strengthens and protects cross-linkage in gluten network (R-S-S-R)	Bromate, dehydroascorbate	[98]
	Preservation of frozen, farmed salmonoids	Protection against early stages of lipid oxidation in raw, frozen fish by in-feed astaxanthin	Antireductant action of astaxanthin	Astaxanthin	[77,98]
	Deferrization and demanganization of drinking water	Protection from precipitate formation and clogging of water pipes on contact with air	Preventive removal of soluble iron (Fe^2+^) and manganese by aeration	O_2_	[78]
Production, biosynthesis	Textile and industrial dyeing	Improvement of colour efficiency during dyeing via protection of fibre reactive dyes from chemical reduction	Sacrificial reduction of antireductant	Ludigol (sodium 3-nitrobenzenesulfonate)	[94]
	Electro-biosynthetic production of chemicals	Cheap energy production (wind turbines, and solar energy captured in photovoltaic cells) powers autotrophic microbial electro-synthesis	Protection of microbiota from reductive stress (electrons) by means of antireductant	H^+^ (H_2_-formation), HCO_3_^−^ (methanogenesis), anode* (electron acceptor)	[37,95,96]
Environment	Protection of primordial life from H_2_	Natural protection from H_2_ inhibition of e.g. fermentation and N_2_-fixation in reducing environment	H_2_ removal by reduction of antireductant, or by energy-yielding respiration of inorganic electron-acceptor as final H_2_ sink	ETC components, exogenous electron acceptors for respiration: carbonate (HCO_3_^−^), sulfate (SO_4_^2−^) up to sulfur (S^0^), nitrate (NO_3_^−^), nitrite (NO_2_^−^), Fe(III)-iron, Mn(III, IV)-manganese, Cr(VI)-chromium, U(VI)-uranium	[30,31,35,38,39]
			Removal of e.g. H_2_ and H_2_S by anoxygenic photosynthesis	Autotrophic CO_2_- and N_2_-fixation	[34]
	Protection of plant rhizosphere	Natural protection of plant roots in water and sediment from reduced, toxic microbial products (H_2_, H_2_S, acids)	Chemical reduction of, or energy-yielding respiration with antireductant	O_2_	[44]
	Protection of photosynthesis from reductive stress (in cyanobacteria)	Natural protection from overproduction of electrons generated by photolysis of H_2_O	Although photosynthesis inhibits respiratory energy production (‘light inhibition of respiration’), photosynthetic ETCs are coupled via mobile plastoquinone to various, membrane-bound terminal respiratory oxidases as sinks for surplus electrons	O_2_ as final electron acceptor	[36]
	Mitigation of ruminal methane emission (greenhouse gas)	Out-competition of methane production in the rumen	Added antireductant as alternative H_2_ (or electron)-sink to methane precursors is energetically more favourable for rumen microorganisms than methanogenesis	Nitrate (NO_3_^−^), sulfate (SO_4_^2−^), fumarate, catechin and resveratrol (O_2_ would lead to feed mineralization instead of valorization)	[23,27,32,33,40,41,91,97]

This review brings together aspects of prokaryotic and eukaryotic cellular physiology, health, production and ecology, in the setting of the multidisciplinary–but often self-contained–foci of antioxidant and antireductant research. Terminology differs in different fields; in this review ‘antioxidant’ is consistently used for electron donor, and ‘antireductant’ for electron acceptor. Natural, plant secondary metabolites and other biogenic molecules are probably most suitable for application in food and feedstuff. Growing environmental and safety concerns stimulate consideration of sustainable biogenic compounds also in non-biological disciplines [5]. Table 1 gives an overview of antireductants and their current or anticipated applications in different sectors. Collation of the existing knowledge about biogenic antireductants in this review might help to draw parallels between disciplines, get enlightened, inspired and pursue innovation.

## ANTIREDUCTANTS FROM THE OUTSET

Although antioxidants form the centre of current attention, it is their counterparts–antireductants–that can be traced back as far as the origin of life. The presumed first role of antireductants was to detoxify the primordial, anaerobic atmosphere by removal of H_2_ [19]. The antireductant scenario thus started during the evolution of the most early metabolic pathways.

A decisive criterion for qualification as an H_2_-sink is its proneness to accept electrons, or–in other words–its susceptibility to chemical reduction. The electrochemical reduction potential, for instance, forms a measure (in volts) of the electron affinity of a putative antireductant. Compounds with highly positive reduction potentials are the most easily reduced substances, and thus represent efficient electron acceptors under both oxygenic and anoxygenic conditions [22]. This principle does not only apply to protective antireductant reactions, but also to energy yielding redox-reactions. The underlying thermodynamic foundation is a linear correlation between the change in standard reduction potentials Δ*E*^0^′ [V] of redox-reactions and the Gibbs free energy change Δ*G*^0^′ [kJ] (adaptable to non-standard conditions by means of the Nernst equation, see below). Thus, in addition to antireductive protection from H_2_, electron transfer from H_2_ to electron-accepting compounds allowed the first organisms to gain energy. Different prokaryotes are able to perform respiration with inorganic electron acceptors, such as carbonate (HCO_3_^−^), sulfate (SO_4_^2−^) up to sulfur (S^0^), nitrate (NO_3_^−^) or nitrite (NO_2_^−^) [30], or with metal (hydr)oxides such as Fe(III)-iron, Mn(III, IV)-manganese, Cr(VI)-chromium or even U(VI)-uranium [31] (Table 1). The more energetically efficient the type of respiration is an organism employs (e.g. O_2_ > NO_3_^−^ > fumarate > SO_4_^2−^ > HCO_3_^−^), the higher its chances to outcompete others by overgrowing them [30,32,33]. Because ranking of electron acceptors by Gibbs free energy change Δ*G*^0^′ (per mole of accepted H_2_) matches their ranking by standard reduction potentials Δ*E*^0^′ [30], reactivities of these electron acceptors as antireductants follow the same ranking order from good to bad.

Next to exergonic redox-reactions, anoxygenic photolysis was an early microbial energy source, with electron donors such as H_2_, hydrogen sulfide (H_2_S), sulfur (S^0^) or Fe(II)-iron [34]. Early forms of respiration as well as sun light provide energy for lithotrophic growth: The respective archaea and bacteria form metastable biomolecules by assimilatory reduction of inorganic compounds such carbon dioxide (CO_2_) and nitrogen (N_2_). Although H_2_ is an electron donor for N_2_-fixation (e.g. in *Clostridium pasteurianum*), additional antireductants to assimilatory N_2_-reduction are needed in an H_2_ atmosphere to protect the enzyme nitrogenase from H_2_-inhibition [35].

Though H_2_ continued escaping from the earth's atmosphere [19], it is currently still being replenished in all living cells as reducing equivalents in the form of nicotinamide adenine dinucleotide (phosphate). NAD(P)H forms the reducing agent, whereas NAD(P)^+^ is the corresponding oxidized form. In general, NADPH is formed for anabolic reactions, for example in photosynthesis, whereas NADH is released during CHO-compound dissimilation and employed to retain energy in the form of ATP. Both photolytic NADPH- and ATP-production, and ATP-yielding respiration make use of electron transport chains (ETC) situated in cell membranes [36,37]. If a microorganism is unable to perform ETC-coupled energy recovery, fermentation forms a–less energy-yielding–alternative [36]. During fermentation, oxidative ATP-formation (via dehydrogenase/kinase reactions) results in excretion of reduced organic products such as lactate, ethanol, formate and H_2_ as final electron-carriers. Hence, although during respiration or photosynthesis H_2_ is oxidized and thus removed, fermentation is another source of H_2_ and other reducing agents [30]. High H_2_ levels are growth-limiting to H_2_-releasing microorganism [38,39], which again emphasizes a need for antireductive, H_2_-lowering mechanisms. Wolfe and co-workers [38] were the first to recognize interspecies-hydrogen transfer and consumption as interdependence in a consortium of microorganisms, enabling the growth of the H_2_ producer. Osburn and Amend [39] showed that the archaeal prokaryote *Thermogladius shockii* WB1 removes fermentatively generated H_2_ by conversion with S^0^ to H_2_S (Table 1). For the anaerobe, H_2_ accumulation obviously forms a bigger problem than H_2_S.

An old attainment, which is widespread among prokaryotes and eukaryotes, seems to be cytosolic or ETC-dependent fumarate conversion. As antireductant, fumarate lowers the emission of the greenhouse gas methane from the upper stomach–the rumen–of cows and sheep [28,40] (Table 1). The rumen is a digestor, in which microorganisms ferment feed to reduced organic compounds and H_2_. In this way, microbes make nutrients from grass and leaves available to the ruminant. In the highly reducing environment of the rumen, fumarate–when added to feed–lowers methane production by acting as alternative H_2_-sink to HCO_3_^−^ [28,40,41]. When fumarate is supplied solely to this reducing environment, fumarate undergoes anabolic reduction, but also catabolic oxidation for energy gain. Excessive reducing equivalents are–under these substrate-limiting conditions–disposed of via methane emission [28]. The feasibility of a mineral-catalysed, photochemical formation of succinate from fumarate, such as in a reverse TCA cycle [42], even suggests a role of fumarate in very first biomolecule formation. Probably, simple organic acids, such as fumarate, and quinones are among the oldest organic antireductants [43].

When O_2_ emerged in the Earth's atmosphere (as a result of the evolution of plant-type photolysis of H_2_O), organism started using O_2_ as an antireductant. Even self-protection of oxygenic photosynthesis includes utilization of O_2_ as relieving electron-sink during reductive stress [36]. In addition, plants still employ O_2_ to protect their root system from reduced, toxic microbial products [44]; and mammals use O_2_ in their colonocytes for H_2_S-removal [45] (Table 1). However, an increasingly oxidizing atmosphere made the development of antioxidant mechanisms imperative, too [19]. Nevertheless, up to the present, antireductive electron-sinks, such as assimilation and respiration are protective of life, and play major roles in global biogeochemical cycling of carbon, nitrogen, sulfur and metals.

Apart from aforementioned molecules, numerous electron-transferring compounds exist in biological systems with different reduction potentials, fulfilling different metabolic needs. Examples are various quinones, nicotinamides, flavins, carotenoids, xanthophylls and other pigments; but also iron–sulfur compounds, and tetrapyrroles coordinated to metals such as cytochromes, chlorophylls or cobalamins (vitamin B_12_) [36,37]. Most electron carriers, redox factors or ETC components are first reduced in their biological function, and re-oxidized upon passing on their electron(s). In the light of the natural, electron-accepting role of these compounds, and depending on their reduction potential, they are probably identical with or have co-evolved with protective antireductants. Reports of ETC-coupled and other protectors from reductive stress [8,36,38,39,46–51] are supportive of an *in vivo* antireductant function of a plethora of biogenic molecules. Moreover, on a cellular level, complex, regulated protection mechanisms against reductive stress seem to exist, analogous to and intertwined with cellular antioxidant pathways: Grant and co-workers [51] were the first to report induction of protein-synthesis protection and of damage control genes in *Saccharomyces cerevisiae* as a cellular response to DTT-induced reductive stress (Table 1). The interest in reductive stress and its role in disease has multiplied over the last five years (PubMed all-fields’ search results for ‘reductive stress’ start from single-digit hits per year in 1987 to two-digit retrievals per year since 2012). However, prevention of or protection from reductive stress is scarcely researched yet (exceptions are presented in Table 1 and below). Nonetheless, not only the health sector, but also other sectors such as nutrition, production and environment are already, and will be, benefitting from antireductants (Table 1).

## ANTIRADICALS: ANTIOXIDANTS AND ANTIREDUCTANTS APART TOGETHER

Most biomolecules are non-radical, metastable compounds, which depend on enzyme-catalysis for their conversion in biological systems. Radicals, by contrast, are more reactive due to the possession of one or more unpaired valence electrons. While dormant radicals form an integrated and controlled part of enzymes in living cells [52], free radicals react spontaneously with different targets. Free radicals are biogenerated for example by leakage of electrons directly to O_2_ during respiration, or by injuries caused through exposure to UV-light, infection or toxin action [11]. On the one hand, free radicals in physiological concentrations act as hormetic stressors and activate repair systems [12], or are even purposively synthesized to counteract pathogens in phagolysosomes, such as the superoxide anion (O_2_^− •^) [53]. On the other hand, highly reactive out-of-control free radicals can cause damage to DNA, proteins and lipids, resulting in mutagenesis or cell death [11,52].

‘Scavenging’ or ‘quenching’ of free radicals is the terminology often used with indirect determination of radical elimination. Basically, chemical mechanisms of radical-clearance comprise electron-transfer reactions and adduct formation [54–57] (Table 2). A one-electron transfer converts free radicals into less-reactive, paired-electron compounds [22]. By means of a one-electron reduction, antioxidants prevent radicals from oxidizing other molecules. Alternatively, free radicals can be oxidized by transferring one electron to an electron acceptor. In that case, this electron acceptor is an antireductant, because it prevents other molecules from becoming reduced by the free radical. Although both modes of action are often invariably termed antioxidation, Martínez et al. [22] specified the difference between the two mechanisms, and united both antioxidants and antireductants acting on free radicals under the term ‘antiradicals’. Radicals can react simultaneously via different mechanisms, and products also depend on which responsive target sites are within closest reach [55,56,58,59] (Table 2). Experimental elucidation of reaction cascades and mechanisms in biological systems still poses a major challenge [14]. Computational modelling provides a tool for calculation of electrochemical quantities, such as the vertical electron affinity of a complex molecule as a measure for its antireduction capacity [14,57,60–62]. However, accuracy of computational prediction depends on the extent to which the complexity imposed by natural conditions can be simulated [61,63]. Hence, due to methodological restraints, the terms antioxidant and antireductant typically refer to electron donation or acceptance in radical research, but not to *in situ* or *in vivo* protective efficacies [12,14].

**Table 2:**
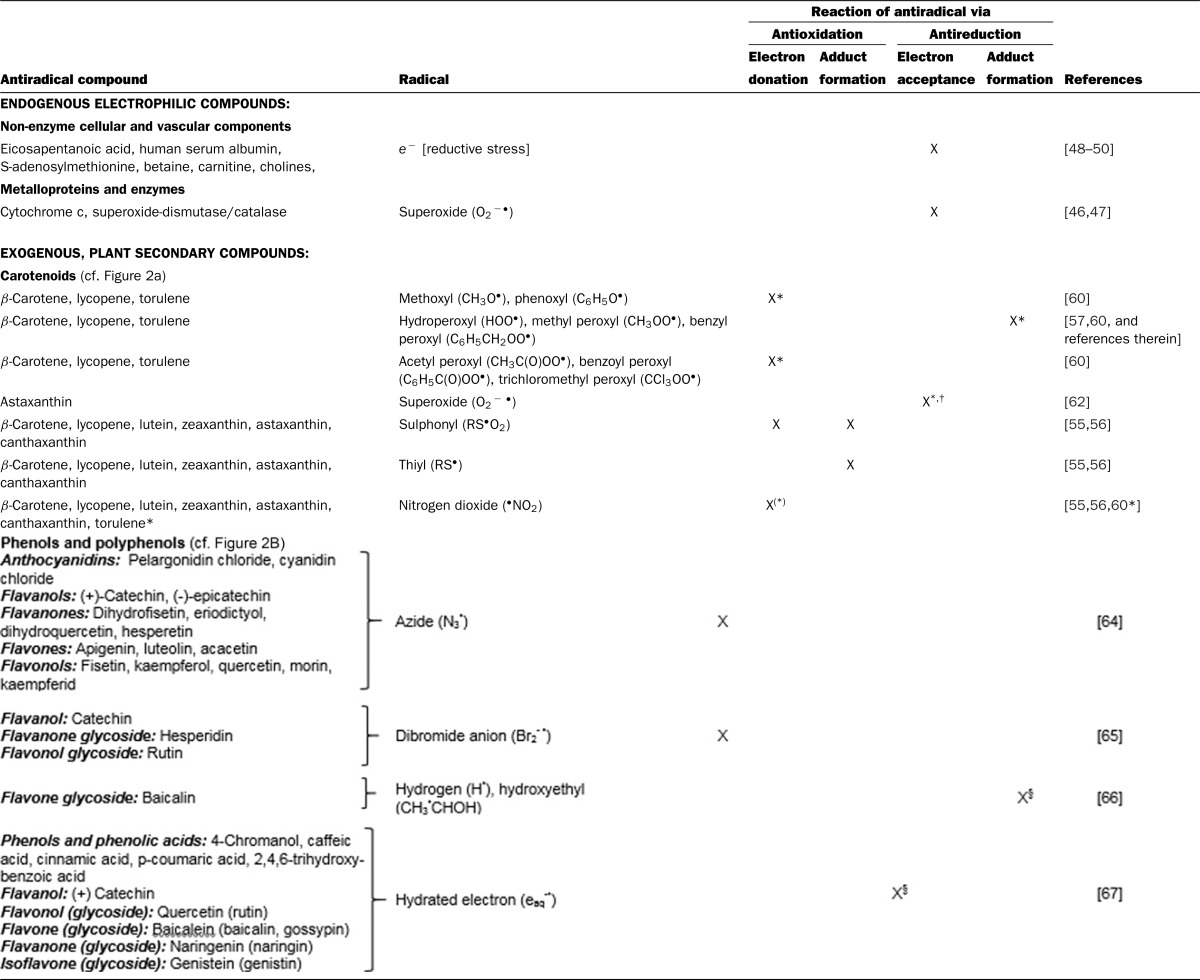
Mechanisms of radical scavenging or quenching, with a focus on antireduction Examples of antioxidation are included for comparison. Indices: *determined by computational modeling; ^†^not in polar solvents; ^§^in O_2_-free aqueous solution.

Natural, polyphenolic compounds, such as flavonoids, were established as electron donors (antioxidants) with highly reactive, electrophilic azide and dibromide radicals (Table 2). Resulting aroxyl radical products were confirmed by means of absorption spectra [64,65]. In contrast with the aforementioned radicals, the superoxide radical was unreactive with such antioxidants [64]. Polyphenolic antireduction was demonstrated under O_2_-free experimental conditions: A number of flavonoids and phenolic acids were reduced by nucleophilic radicals, such as hydrated electrons (e_aq_^− •^) [64,66,67], the hydrogen radical (H^•^) [66] and formate radical (CO_2_^−^) [64]. The 1-hydroxyethyl (CH_3_^•^CHOH) radical also acted as a reducing agent in the absence of O_2_, implying antireduction [66].

With carotenoids, extensive computational modelling has been performed to predict their reactivities with free radicals. Oxygen-centred radicals, such as methoxyl and phenoxyl radicals are supposed to withdraw electrons from carotenoid antioxidants in aqueous phase under standard conditions [57] (Table 2). Peroxyl radicals with an electron-withdrawing group (benzoyloxy, acetyloxy or trichloromethyl) are predicted to also act in this manner. By contrast, peroxyl radicals with electron-releasing groups (hydrogen, methyl, benzyl) are expected to form adducts with antireductants such as the carotenoid torulene [57,60] (Table 2). Antireduction is also the suggested way of clearance of the negatively charged superoxide radical, produced by oxidases in cells and blood vessels [47]. Antireduction as mechanism of action is in line with the relatively low standard reduction potential *E*^0^′ (of −0.16 V) of superoxide generation from O_2_ [68], its ability to reduce Fe^3+^ [54,68] and cytochrome *c* [46,47,54], and the calculated capacity to reduce the carotenoid astaxanthin in benzene [62]. In various organisms, the superoxide radical is cleared by superoxide-dismutase; i.e. superoxide is alternately reduced to H_2_O_2_ and oxidized to O_2_. This enzyme has therefore been classified as both antioxidant and antireductant [47] (Tables 1 and 2).

**Figure 1:**
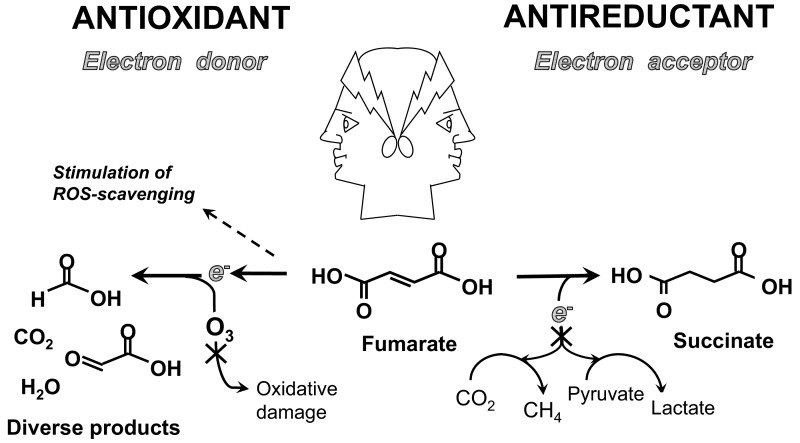
Janus-faced compound fumarate: both antioxidant and antireductant in one molecule Sources of data: [40,74,83,84].

Radicals of xenobiotic origin, such as the chlorine radical (disinfectants), the 1-hydroxyethyl radical (alcohol consumption) or the nitrogen dioxide (^•^NO_2_) radical (cigarette smoke), initiate lipid autoxidation on their exposure to unsaturated fatty acids [55,58,69,98]. Lipid autoxidation contributes to deterioration of fats and oils, and damages in biological membranes [8]. Primary attack to a C═C double bond by electrophilic radicals results in alkyl radical formation. If a carbon-centred radical is not preventively cleared by antireduction, it reacts with O_2_ to an alkyl peroxide (ROO^•^). This peroxyl radical then attacks another alkene double bond, producing another alkyl radical, and thus starts an autoxidative chain reaction [54]. Antireductants, which react with alkyl radicals, hence must be able to compete with O_2_ for electron acceptance [8]. There are antireductants, which are stable in the presence of O_2_, such as quinones [8,47], or the commercially available galvinoxyl radical [70]. Nevertheless, in view of the high standard reduction potential *E*^0^′ of the redox couple O_2_/H_2_O of +0.82 V [71], antireduction seems to work best in anoxic environments [8,19,23,27,66,67]. Then again, in most cells and tissues, reducing capacities are high and O_2_ levels low [18,72,73]. Probably due to respiration and endogenous antioxidation, intact biological systems are highly self-controlled and–regulated [52]. While in animals and man, reducing cofactors such as NADH determine the intrinsic redox status, in fruits and vegetables ascorbate and plant phenols are important redox determinants [64]. The redox status inside living matter is measured as apparent reduction potential *E*_h_ of redox couples following the Nernst equation (*E*_h_=*E*^o^′ + (*RT*/*nF*) × ln([Ox]/[Red]), with *E*^o^′: reduction potential at standard conditions, *R*: gas constant, *T*: temperature, *n*: number of moles of electrons transferred, *F*: Faraday's constant). This equation factors the ratio of oxidized to reduced molecules of e.g. glutathione or cysteine [RSSR]/[RSH]^2^, as *in situ* coexisting concentrations. In yeast cytoplasm, glutathione [RSSR]:[RSH] ratios of 1:∼70–190 were reported [51]. For mammals, Go and Jones [73] compiled glutathione-related *E*_h_ values of approximately −0.2 V in red blood cells (as oxygen carriers) and the lung lining fluid, less than −0.2 V in cells and tissues, and approximately −0.3 V in mitochondria, the organelles of dissimilatory NADH-generation and aerobic respiration. In view of these reducing conditions, endogenous antioxidation and antireduction probably complement one another and thus coexist. The part antireduction plays in redox homoeostasis becomes obvious during phases of O_2_-depletion, or an unbalance of the cellular [NAD^+^]:[NADH] ratio. For instance, exogenous fumarate had a cardioprotective effect during hypoxia, as it acts as competitive electron acceptor to endogenous, necrotizing lactic acid fermentation [74] (Figure 1). Elevated dissimilatory NADH levels can result from high blood glucose levels, such as during persistent over-nutrition. The thus caused reductive stress was reported to manifest itself as enhanced electron leakage and formation of oxygen radicals during respiration. The oxygen radicals are then the source of oxidative stress [50,75,76]. Early protection from reductive stress is offered by diverse endogenous antireductants, such as serum albumin (via disulfide reduction), and biomolecules with positively charged, methylated N or S atoms [49,50] (Tables 1 and 2).

Exogenous antireductants against nucleophilic radicals in living systems can only play a physiological role at sites where they can be made bioavailable. In human beings, conceivable areas of application are the skin [2], the intestinal epithelium as entry port of in-food contraries and pathogens, followed by the blood stream, up to body fat, in which lipophilic compounds accumulate [77]. In view of the high reactivity of free radicals, large amounts of antireductants might be necessary for local, competitive protection [12]. However, exogenous antireductants, as oxidizing agents, must not unbalance the delicate redox homoeostasis: On overdosing, an antireductant might become an inducer of oxidative stress (commonly referred to as pro-oxidant). Electrophilic therapeutics, intended to activate cellular antioxidant mechanisms, were shown to cause oxidative modifications to other molecules and sustain activation of reducing pathways (e.g. KEAP1–NRF2 [*Kelch-like ECH-associated protein 1–nuclear factor (erythroid-derived 2)-related factor 2*] disulfide reduction) [29]. In excess, antireductants might, as electrophilic therapeutics, hyperactivate responding systems, and similarly cause proteotoxicity and further damage [29]. Hence, as with antioxidants for health promotion [11,12,15,17,29], antireductant application seems equally challenging. Nevertheless, in a slightly different context, the strategy seems to work: In-feed astaxanthin was shown to become incorporated in salmonid fat during aquaculture, and was inferred to act as a food-preserving antireductant in the subsequently frozen fish [98] by provision of protection against early lipid oxidation [77] (Tables 1 and 2). Undoubtedly, anoxic environments [8,19,23,27,66,67,74] and interphases between anoxic and oxygenic settings [44,45,78,98] encourage antireductant operation (Table 1).

In terms of active compounds, current data indicate that apart from electronegativity, the impact of functional groups (on electron density, delocalization and affinity), as well as accessibility of active sites to both radical and antiradical are crucial for the type of reaction between them [59,61,79]. In addition, the reaction environment (polarity, temperature, pH) and actual concentrations of all reactants represent important factors, because normal life conditions are non-standard conditions [12,37,58–60,63,67,72]. Taking these factors into account, the susceptibility of a radical to antireduction seems to basically depend on its nucleophilicity, and the difference between the non-standard reduction potentials of the combined half-reactions as driving force [22,61,79,80].

## JANUS-FACED COMPOUNDS: BOTH ANTIOXIDANT AND ANTIREDUCTANT IN ONE MOLECULE

In many antioxidants, antireductant function has been predicted or discovered [14,22,23,27,60,67]. The compounds in question can both donate electrons as antioxidants (and being themselves oxidized), and accept electrons as antireductants (and being themselves reduced). These properties furnish them with a dual, Janus-faced nature. For the sake of clarity, ‘dual nature’ does not refer to redox couples, and hence neither to regenerating actions as described for vitamin E, vitamin C, glutathione, etc. [79,81], nor to *in vivo* redox-signalling or -regulation via either direct reduction or oxidation [12,82]. On the contrary, starting with one and the same compound, either antioxidant, or antireductant action generates either oxidized or reduced conversion products. Well-known natural antioxidants such as certain carotenoids [14,22,56] and flavonoids [23,27,67] fall into this category, but also rather small metabolites as fumarate [40,74,83,84] (Figure 1). This section focuses on Janus-faced compounds and their biochemical conversions as antioxidants and antireductants. Possibilities of reactivity enhancement are specified for antireductants.

**Table 3:** Janus-faced compounds: natural antioxidants as antireductants (with respective reactivities depending on polarity, pH and O_2_-level of the setting) Abbreviations: AR, antireductant; CM, computational modelling of electron affinity of AR as electron acceptor in polar solvent; ECR, experimental chemical reduction of AR under O_2_-free conditions; EMR, experimental microbial reduction of AR as alternative hydrogen sink to methane precursors under anaerobic conditions.

Antireductants	Setting	References
**Primary metabolite:** Fumarate	EMR	[40]
**Carotenoids:** β-Carotene, lutein, zeaxanthin, echinenone, canthaxanthin, adinorubin, astaxanthin	CM ECR	[22,88] [14]
**Psittacofulvins:** Tetradecahexenal, hexadecaheptenal, octadecaoctenal, eicosanonenal	CM	[88]
**Anthocyanins:** Peonidin, cyaniding, delphinidin, pelargonidin, petunidin, malvidin	CM	[88]
**Phenols and flavonoids:** (+)-Catechin, 4-chromanol, genistein, genistin, rutin, caffeic acid, *trans*-cinnamic acid, *p*-coumaric acid, 2,4,6-trihydroxyl-benzoic acid, baicalein, baicalin, naringenin, naringin, quercetin, gossypin	EMR ECR	[23,27] [67]

**Figure 2:**
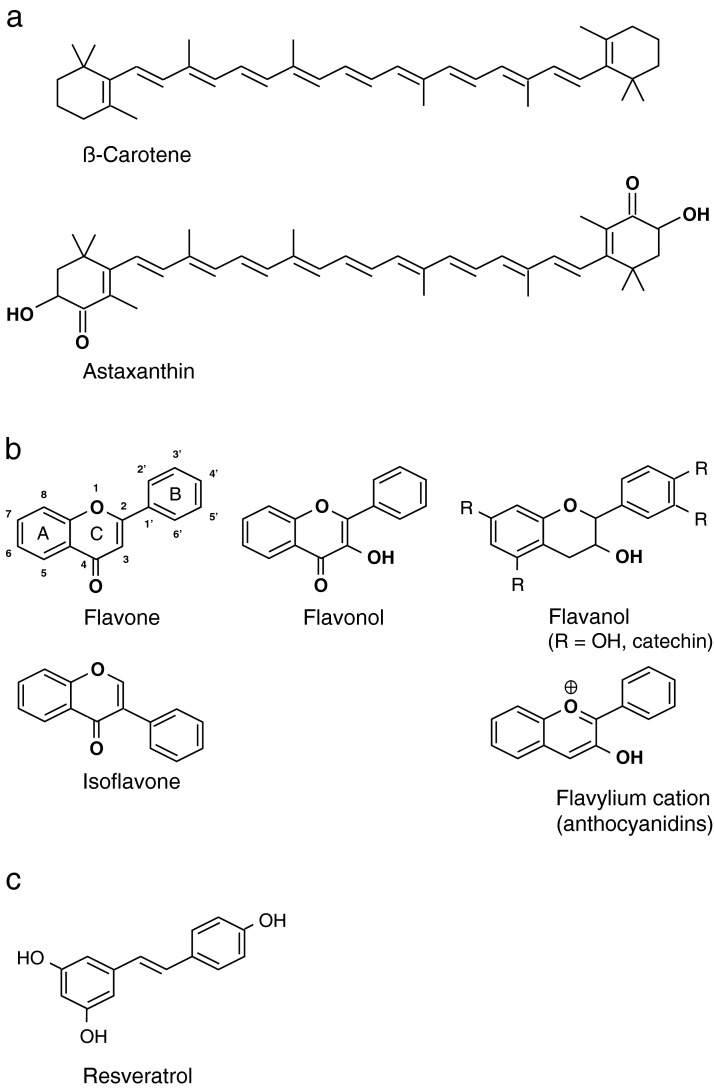
Chemical structures of different plant secondary compounds, among them classical antioxidants and acknowledged antireductants **(a**) Carotenoids; (**b**) backbones of flavonoids (derivatives, such as catechin or anthocyanidins, carry e.g. hydroxy groups in different positions of the backbone); (**c**) resveratrol, a stilbenoid.

Carotenoids, i.e. plant secondary compounds such as β-carotene, zeaxanthin and lutein, have been reported to act both as antioxidant, and as antireductant under respectively suitable conditions [14,22,85] (Table 3). Antioxidant reactions occur at various double bonds of the conjugated backbone of carotenoids (Figure 2a). β-Carotene, for example, is oxidatively cleaved during antioxidation into diversely-sized fragments with hydroxy, carbonyl, carboxy and epoxy groups [17]. Both antioxidation and antireduction capacities of carotenoids do not seem to be affected by the isoprene-derived methyl groups of their polyene backbone. Calculated data for compounds with 4 and 9 conjugated double bonds in their polyene chain were in line with those for the equally alkenylated, but in addition methylated vitamin A (retinol) and β-carotene (Figure 2a) [22,85]. By contrast, the size of the conjugated system is pivotal for the electron-capturing antireduction capacity, as substantiated experimentally and computationally: Larger polyene molecules with a more extended conjugated system represented better antireductants than smaller ones [14,85]. Keto (C═O) group(s) on the terminal ring(s), which prolong the conjugated system, equip carotenoids with a higher electron-accepting, antireduction potential. A hydroxy group next to the C═O group, such as in adinorubin or astaxanthin (Figure 2a), again seems to enhance antireductant quality [22]. As a side effect, oxygen substituents make lipophilic carotenoids better soluble in aqueous solution [79]. The antireductant capacity of natural carotenoids can be improved by chemical modification: Martínez [86] showed by electron density modelling that exchanging the C═O groups, as of canthaxanthin, by C═S, C═Se or C═Te enhanced the antireductant potential of the parent compounds. Another option to improve electron acceptance is to make use of the metal-complexing ability of highly oxygen-substituted carotenoids. Metal chelation by astaxanthin, via interaction of divalent metal cations with oxygen atoms of an end ring's C═O group and deprotonated hydroxy group, resulted in higher electron-accepting capacities. Concurrently, the resulting chelate also showed increased electron-donating, i.e. antioxidant, capacities [87]. Remarkably, the removal of metal ions in itself by complexation has been associated with prevention of oxidative stress [4,87].

Psittacofulvins are non-carotenoid polyenes with one terminal carbonyl group. Psittacofulvins occur only in parrot feathers, and they are of special interest as model compounds for comparative electron affinity computation. In a non-polar environment (benzene), they were calculated to act as electron-donating antioxidants, whereas in water they would attract electrons as antireductants [88] (Table 3). Psittacofulvins–as an outlier here–offer a glimpse of the huge variation, and variability, of biogenic compounds as basis for multifunctionality in nature. Apart from being classified as antioxidants or antireductants, many plant secondary compounds are also pigments, absorbing light for energy recovery or provision of colour (even after being eaten), or–alternatively–are plant-protective feeding-repellents [64].

Flavonoids such as anthocyanidins (Figure 2b) exist in different stable configurations at different pH values. At low pH, they are positively charged, and at high pH, they are negatively charged. According to computational modelling, neutral or negatively charged anthocyanidins make good electron-donating antioxidants. Positively charged anthocyanidins are very effective electron-accepting antireductants [88] (Table 3). In the related flavones (Figure 2b), antioxidation is mainly provided for by the C-ring of the molecule, namely by the double bond between carbons 2 and 3 and the keto group on carbon 4. In flavonols (Figure 2b), the added hydroxy group on carbon 3 in the C-ring increases antioxidant activity [89]. In flavanols (Figure 2b) with their saturated bond between carbons 2 and 3 in the C-ring, the site most prone to one-electron donation is a deprotonated (negatively charged) hydroxy group on carbon 4′ in the B-ring, assisted by an *ortho*-positioned, second hydroxy group [64,90]. Aroxyl radical species as primary oxidation products are stabilized by extensive electron delocalization [64]. As antireductant, the flavanol (+)-catechin (Figure 2b) proved step-wise degraded; C-ring opening was followed by A-ring cleavage. The hydroxy groups on carbons 5 and 7 of the A ring–which are oxidized during antioxidation [64,90]–together with the heterocyclic oxygen of the C-ring, also ended up as aliphatic carboxylic or keto group after ring fissions under reducing conditions [23]. However, neither oxidation, nor reduction was involved during (anti)reductive C- and A-ring cleavages, but chemical rearrangements and hydrolyses. Product identification revealed that (anti)reductive conversions comprised essentially diverse hydrogenation reactions [23]. The B-ring of (+)-catechin–most susceptible to oxidation at its hydroxy group on carbon 4′ [64,90] (see above)–was the last ring present as identifier of the parent compound during (anti)reductive catechin degradation. Although catechin conversion proceeded via different reaction sequences in rumen fluid, the B-ring of (+)-catechin was shown to lose its hydroxy group on carbon 4′ at some point in all of the sequences by reductive dehydroxylation [23].

The stilbenoid resveratrol (Figure 2c) is a good electron-donating antioxidant, but a bad electron accepting antireductant, according to its positioning on a donor–acceptor map (relative to melatonin and α-lipoic acid by means of calculated donation and acceptance indices) [26]. Despite this ranking, under anaerobic conditions, resveratrol is reduced as an antireductant when simultaneously lowering methane emission [27,91] (Tables 1 and 3). Evidence was obtained for hydrogenation of the aliphatic double bond of resveratrol on its (anti)reductive conversion [27].

Interestingly, bioreduction of the flavanol catechin (Figure 2b) and of the stilbenoid resveratrol (Figure 2c) was first discovered during research focusing on the low bioavailability of these antioxidants after oral ingestion. It became evident that gut microorganisms degrade these plant antioxidants to reduced metabolites [92,93]. Although the loss of supposedly health-promoting antioxidants by microbial reduction in the digestive tract is obviously undesired, the same conversions were shown to lower emission of the greenhouse gas methane from the upper stomach of ruminants [23,27,91]. Greenhouse gas mitigation is a contemporary requirement in the framework of global climate change. As competitive hydrogen-sinks, catechin and resveratrol removed H_2_ as by-product from rumen fermentation and thus facilitate plant material digestion. Hence, in the rumen those natural compounds function as hydrogen- and methane-lowering antireductants. They prevent biochemical reduction of HCO_3_^−^ to methane by undergoing competing, reductive reactions themselves [23,27]. Not only acknowledged natural antioxidants such as catechin and resveratrol function in this way [23,27], but also small compounds such as fumarate [40,41] (Tables 1 and 3).

Fumarate, as an exogenous antireductant, not only lowered ruminal methane emission [40,41], but also protected the heart under O_2_-deficiency [74] (Table 1). As antioxidant, fumarate was reported to activate an enzyme (GPx1) that scavenges ROS [84], and to bind ozone, for example [83]. In summary, fumarate can either act as an electron-accepting antireductant [40], undergo photochemical or enzymatic reductive assimilation [28,42], act as an energy and electron source [28], or play diverse roles in antioxidation [83,84]. All its capacities make fumarate not just a paradigm of a Janus-faced antioxidant/reductant (Figure 1), but a truly multifunctional compound.

## CONCLUDING REMARKS

Up to now, antireductants appeared only sporadically in scientific papers. The sparse pieces of information available on antireduction were comprehensively gathered from diverse research disciplines (Table 1) for this review. Hence, this review substantiates–for the first time–that antireduction is an ancient strategy, that it is still essential for current life, and that it offers promising perspectives for future applications. Cellular ‘redox homoeostasis’, for example, can only be maintained under natural protection by both antioxidants and antireductants. Moreover, seemingly unrelated topical fields, such as ‘reductive stress’, ‘electro-biosynthesis’ or ‘mitigation of ruminal methane emission’ all relate to antireduction (Table 1), and might profit from the knowledge collated in this review.

## Abbreviations

DTT: dithiothreitol
ETC: electron transport chain
KEAP1–NRF2: Kelch-like ECH-associated protein 1 - nuclear factor (erythroid-derived 2)-related factor 2
NAD(P): nicotinamide adenine dinucleotide (phosphate)
TCA: tricarboxylic acid
Tsa1: thiol-specific antioxidant protein 1
